# A high-throughput phenotyping assay for precisely determining stalk crushing strength in large-scale sugarcane germplasm

**DOI:** 10.3389/fpls.2023.1224268

**Published:** 2023-07-20

**Authors:** Fumin Ma, Yinjuan Shen, De Su, Muhammad Adnan, Maoyao Wang, Fuhong Jiang, Qian Hu, Xiaoru Chen, Guanyong He, Wei Yao, Muqing Zhang, Jiangfeng Huang

**Affiliations:** ^1^ State Key Laboratory for Conservation and Utilization of Subtropical Agro-bioresources, Guangxi Key Laboratory of Sugarcane Biology, Ministry Co-sponsored Collaborative Innovation Center of Canesugar Industry, Academy of Sugarcane and Sugar Industry, College of Agriculture, Guangxi University, Nanning, Guangxi, China; ^2^ Guangxi China-ASEAN Youth Industrial Park, Chongzuo Agricultural Hi-tech Industry Demo Zone, Chongzuo, Guangxi, China

**Keywords:** sugarcane, lodging resistance, mechanical strength, crushing strength, NIRS

## Abstract

Sugarcane is a major industrial crop around the world. Lodging due to weak mechanical strength is one of the main problems leading to huge yield losses in sugarcane. However, due to the lack of high efficiency phenotyping methods for stalk mechanical strength characterization, genetic approaches for lodging-resistant improvement are severely restricted. This study attempted to apply near-infrared spectroscopy high-throughput assays for the first time to estimate the crushing strength of sugarcane stalks. A total of 335 sugarcane samples with huge variation in stalk crushing strength were collected for online NIRS modeling. A comprehensive analysis demonstrated that the calibration and validation sets were comparable. By applying a modified partial least squares method, we obtained high-performance equations that had large coefficients of determination (*R^2^* > 0.80) and high ratio performance deviations (RPD > 2.4). Particularly, when the calibration and external validation sets combined for an integrative modeling, we obtained the final equation with a coefficient of determination (*R^2^*) and ratio performance deviation (RPD) above 0.9 and 3.0, respectively, demonstrating excellent prediction capacity. Additionally, the obtained model was applied for characterization of stalk crushing strength in large-scale sugarcane germplasm. In a three-year study, the genetic characteristics of stalk crushing strength were found to remain stable, and the optimal sugarcane genotypes were screened out consistently. In conclusion, this study offers a feasible option for a high-throughput analysis of sugarcane mechanical strength, which can be used for the breeding of lodging resistant sugarcane and beyond.

## Introduction

In crops, lodging is one of the major problems that affect growth and potential yield (Guo et al., 2021). Generally, stalk lodging and root lodging constitute the two most common forms of lodging (Zhang et al., 2016). The term root lodging refers to the entire plant falling to the ground without being bent by the stalk, whereas stalk lodging refers to the stalk inclines and bends at different angles (Berry et al., 2003).

As one of the most commonly grown C4-type industrial crops, sugarcane (*Saccharum* spp.) is known for its high photosynthesis efficiency and high yield (Swapna and Kumar, 2017). However, due to its stalk-harvesting nature, sugarcane faces a much higher risk of lodging, which results in a huge decrease in yield, as well as difficulties with mechanical harvesting, increasing the cost of production (van Heerden et al., 2015). It has been documented that sugarcane lodging is influenced by the environment and phenotype, as well as number of canopy leaves, planting depth, center of gravity height, and stalk hardness (Park et al., 2005; Babu et al., 2010; van Heerden et al., 2010). Specifically, from the perspective of genetic bias, mechanical strength appears to be the most important factor affecting stalk lodging resistance (Xie et al., 2022). It has been shown that stalk mechanical strength can be used as an important index to predict lodging risk, and that bending strength and rind penetrometer resistance (RPR) can reflect stalk mechanical strength (Zhang et al., 2019). A combination of crushing strength, rind penetrometer resistance (RPR), and bending strength has been used to determine the relationship between stalk mechanical strength and lodging (Stubbs et al., 2020; Wang et al., 2020; Shao et al., 2021). In a recent study, we have demonstrated that rind penetrometer resistance (RPR) and breaking force can be used to determine the mechanical strength of sugarcane stalks (Shen et al., 2021). However, it is important to realize that rind penetrometer resistance (RPR) alone cannot properly reflect stalk lodging resistance because it ignores the contributions from the stalk’s cross-sectional area and vascular bundles (Robertson et al., 2016). Particularly, laboratory-based mechanical phenotyping requires a significant amount of time and therefore cannot be used for large-scale genetic screening projects. Hence, it is essential to develop high-throughput assays for measuring the stalk mechanical strength of sugarcane onto a global scale.

The near infrared spectroscopy (NIRS) is a very efficient method that has been widely used for high-throughput determine various chemical and biochemical structures of agricultural crop (Washburn et al., 2013). For instances, NIRS has been used for high-throughput predicting fiber and nutrient content of dryland cereal cultivars (Brenna and Berardo, 2004; Stubbs et al., 2010), phenotyping of moisture and amylose content in maize (Wang et al., 2019; Dong et al., 2021), evaluating the composition of carbohydrates in soybean (Leite et al., 2020; Singh et al., 2021), detecting biomass of plant root mixtures (Roumet et al., 2006), analyzing available P contents in soils to aid fertilization (Patzold et al., 2020), as well as determining the internal quality and physiological maturity in the fruit (Cunha et al., 2016; de Carvalho et al., 2019; Minas et al., 2021). In our previous studies, the NIRS has been successfully applied for stalk quality determination (Wang et al., 2021), cell wall features and lignocellulose digestibility characterization in sugarcane (Li et al., 2021; Adnan et al., 2022). Notably, in a recent study, we have also successfully implemented the NIRS for assessing the mechanical strength of sugarcane stalks by measuring the rind penetrometer resistance (RPR) and breaking force (Shen et al., 2022).

As a coupled complementary exploration, this study aimed to establish a set of methods for high-throughput phenotyping of sugarcane crushing strength. Due to the large number of diverse sugarcane germplasms collected, a precise online NIRS assay was developed using chemometric analysis. After three years of testing in large-scale sugarcane germplasm, the NIRS model exhibited stable and reliable performance, enabling the optimal germplasm to be selected. Therefore, this study provided a reliable strategy for crushing strength determination, which could be integrated with our previous studies for lodging resistant aimed precision breeding in sugarcane.

## Materials and methods

### Experimental site and sugarcane planting

This experiment was conducted at the Fusui experimental field located at Guangxi University (107° 47′17.66′′ E, 22° 31′ 5.85′′ N), and the soil type is loam. As a subtropical monsoon climate, there are 1050 - 1300 mm of precipitation annually, a mean annual temperature of 21.3 - 22.80°C, and a mean annual sunshine of 1693 hours (data source: http://www.gxcounty.com/pindao/112287/). We utilized a randomized block design to plant the sugarcane genotypes at three identical experimental field plots of 5 m row length, 2 m row spacing, and 0.6 m depth. A total of 860 sugarcane germplasm collected from all over China were planted in each planting plot, of which 416 core germplasm samples were selected for crushing strength characterization in this study. All sugarcane germplasm were planted in May 2019 with basal fertilizer (organic-inorganic fertilizer 12-6-7, 750 kg ha^-1^), tillering fertilizer (NPK 20-10-10, 300 kg ha^-1^) and jointing fertilizer (NPK 20-10-10, 1500 kg ha^-1^). For the fertilization of ratoon sugarcane, urea (150 kg ha^-1^) and KCl (150 kg ha^-1^) were applied in April and August, and compound fertilizer (NPK 15-15-15, 1875kg ha^-1^) was applied in May. Pest control was not applied throughout the growing period, but irrigation and weeding were performed as necessary.

### Assay of stalk crushing strength in sugarcane population

An electronic universal testing machine, DNS-20 (Sinotest Equipment Co., Ltd, China), was used to measure the stalk crushing strength. For each sugarcane genotype, the 15^th^ internode was selected to measure stalk crushing strength (kN) (Shen et al., 2021). In summary, the sugarcane stalk was arranged horizontally on the stage to permit direct compression of the internodes by a circular probe of 90 mm diameter. The movement of probe consisted of four processes (descent, gap elimination, compression, lifting). In order to maximize test efficiency and data accuracy, we set the speed of the four processes at 500 mm/min, 150 mm/min, 150 mm/min, 500 mm/min, respectively. The load cell collected force data every 100 ms. Three biological replicates were performed for each genotype planted in each experimental field plot. The mechanical data were recorded and analyzed using the TestExpert software (version 3.2).

### Online NIRS data collection

During the maturity period, 383, 368, and 376 genotypes of sugarcane germplasm were collected from three planting plots in November 2021 for online NIRS modeling. The online NIRS data were collected by a well-established method previously described by Li et al. (2021) with minor modification. Briefly, for each genotype, three plants were randomly selected, and leaves and young tips were removed and immediately shredded using DM540 (IRBI Machines and Equipment Ltd, Brazil). The shredded sugarcane sample was blended and transmitted *via* CPS (Cane presentation system, Bruker Optik GmbH, Germany). The spectral was collected through MATRIX-F (Bruker Optik GmbH, Germany) online system. A full scanning mode was used to scan the shredded samples, with a wavelength range of 4000 to 10000 cm^-1^ in 4 cm^-1^ steps. The absorbance values of the spectra were recorded in log(1/R), where R is the reflectance of sample. To provide a more comprehensive analysis, the OPUS software automatically averaged the online reflectance values obtained. A standard equipped in Q413 sensor of MATRIX-F was scanned every one hour for instrument correction.

### NIRS pretreatments and modeling

The spectral data were collected and analyzed using the OPUS software. Before modeling, the samples were randomly divided into calibration and validation sets in a roughly 4:1 ratio, which was used for modeling and external validation, respectively. Pretreatment of spectral data was performed in order to minimize the risk of physical disturbance. To obtain the optimal spectral region for modeling, OPUS software used ten spectral pretreatment methods in combination to divide the NIRS spectrum into multiple sections (Wang et al., 2021), including constant offset elimination (COE), straight-line subtraction (SSL), standard normal variate (SNV), Min-Max normalization (MMN), multivariate scattering correction (MSC), first derivative (FD), second derivative (SED), combination of the first derivative and straight-line subtraction (FD+SSL), standard normal variate (FD+SNV), and multiplicative scattering correction (FD+MSC). A principal component analysis (PCA) of the raw spectral data was conducted to determine the distribution of spectral groups, and outlier samples were excluded based on GH values (> 3.0). Based on the partial least squares (PLS) method, the calibration equations were generated by combining the selected samples with the optimal parameters. A default setting in OPUS software was used to select the wavelength range. A combination in terms of wavelength range selection and spectrum pretreatment was made to obtain calibration models in PLS analysis (Li et al., 2021; Adnan et al., 2022). Internal cross-validation and external validation of the equations were used to evaluate the performance of the model (Williams and Sobering, 1996). Finally, the optimal equation was selected based on high coefficient of determination (*R^2^*_c_/*R^2^*_cv_/*R^2^*_ev_), ratio of prediction to deviation (RPD), and low root mean square error (RMSEC/RMSECV/RMSEP) from calibration/internal cross-validation/external validation.

### Application of the model in sugarcane population

A total of 336 samples of sugarcane were harvested at maturation in three years (2019, 2020, and 2021), the NIR spectra were online collected as described above. Based on our established model in the year of 2021, the acquired spectral data was analyzed with the help of the OPUS software to obtain the predicted stalk crushing strength across these three years. Samples with GH > 3.0 were considered outlier based on principal component analysis. After excluding all outliers, sugarcane germplasm with high and low stalk crushing strength was screened out.

## Results

### Accurate determination of stalk crushing strength in sugarcane

**Figure 1:**
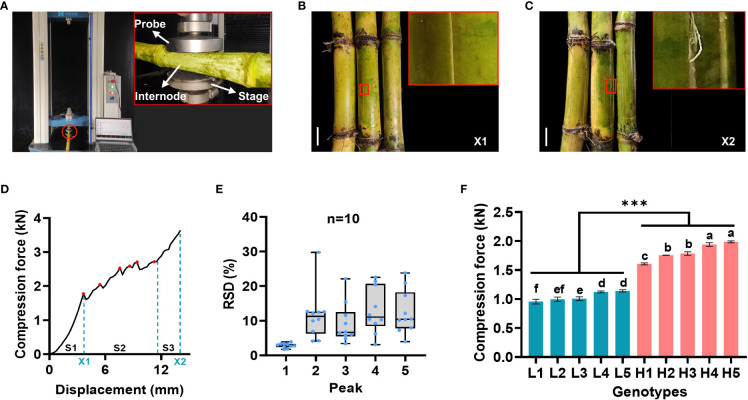
Laboratory analytical method for stalk crushing strength determination in sugarcane. **(A)** Schematic diagram of sugarcane crushing strength determination. **(B, C)** Morphological changes of internode at the moment of cracks appeared **(B)** and complete ruptured **(C)**, bars = 3 cm. **(D)** Compression force curve with multiple peaks for crushing strength determination. S1-S3: three compressive states (elasticity, yield, compaction strengthening); Red dots represent the detected peaks; X1 and X2 represent the key steps as described in B and C, respectively; **(E)** Comparative analysis of each detected peaks in the compressive force curves in ten representative sugarcane samples. RSD: relative standard deviation. **(F)** Comparative analysis of the first peak between two groups of ten representative sugarcane genotypes. Different letters indicated statistically significant differences among these genotypes *via* one-way ANOVA and LSD test at α ≤ 0.05 level; *** indicated statistically significant different between the two groups at *p* < 0.001 level. H1-H5 and L1-L5 represented five sugarcane genotypes with high (H) and low (L) mechanical strength, respectively. Each sample contained three biological replicates.

For a precise and reliable determination of stalk mechanical strength, the 15^th^ internode of the sugarcane stalk was selected to determine crushing strength at maturity. In detail, the selected internode was placed horizontally in the middle of the stage and compressed by a probe with a threshold force of 4 kN (**Figure 1A**). As illustrated in **Figure 1B**, when a certain amount of pressure is applied to the cane stem, cracks begin to appear along its axis. The internodes ruptured when a continuous compressive force was applied to the internodes up to the threshold, causing irreversible morphological changes (**Figure 1C**). In the course of this process, TestExpert software generated a compression force curve with multiple peaks (**Figure 1D**). Remarkably, the curve showed three compressive states (elasticity, yield, compaction strengthening) (Sun et al., 2022). For the purpose of verifying the reliability of each peak, ten randomly selected sugarcane samples were tested for compressive force. A similar fluctuating change in the compressive force between the same samples was observed (**Figure S1**), consistent with the results observed in maize (Kovacs and Kerenyi, 2019; Zhang et al., 2020). Noteworthy, the relative standard deviation (RSD) value of the first peak was significantly less than that of the other peaks (**Figure 1E**; **Table S1**).

Further tests were conducted on two representative groups of ten representative sugarcane genotypes, and the differences in compressive force was clearly observed between them (**Figure S2**). Particularly, a comparison of the first peak of compressive force in the two groups revealed that there was a significant difference between them (**Figure 1F**), indicating that the first peak was sufficient to identify the high and low samples. Therefore, it was appropriate to assess the crushing strength of sugarcane stalks based on the first peak.

### Diversity of stalk crushing strength in sugarcane population

**Figure 2:**
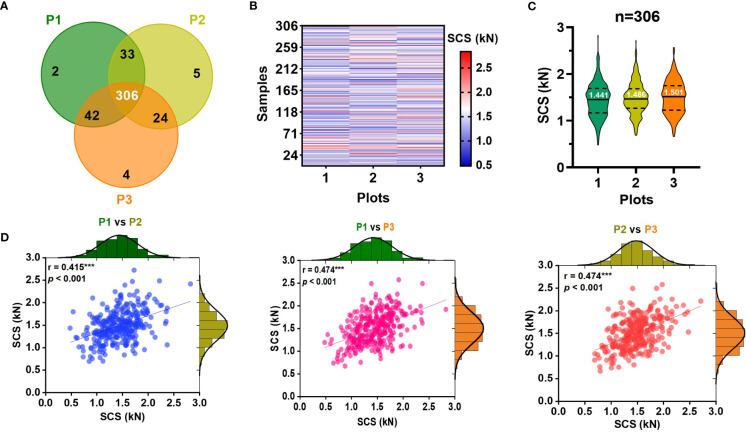
Diversity of stalk crushing strength (SCS) in collected sugarcane samples. **(A)** Venn diagram of sugarcane samples collected from three identical experimental field plots. **(B)** Heatmap and **(C)** violin chart displaying the stalk crushing strengths in collected sugarcane genotypes. **(D)** Distribution and correlation analysis of stalk crushing strength of 306 sugarcane genotypes in three planting plots. *** indicated significant correlations at *p* < 0.001 level. P1-P3: three planting plots.

Sugarcane germplasm planted in three experimental field plots were applied for the stalk crushing strength determination by DNS-20 electronic universal testing machine. In detail, 383, 368, and 376 sugarcane samples were harvested at maturity in each of the three planting plots and the first peak from the compressive force curves was recorded (**Figure 2A**). Among these samples, 306 were common to all three plots and they displayed a wide range of agronomic trait variability (**Table S2**). Specifically, their crushing strength exhibited considerable variation, although some genotypes varied across three planting plots (**Figures 2B, C**; **Table S3**). An analysis of correlations revealed that stalk crushing strength was negatively correlated with internode length, but positively correlated with stalk diameter and internode number (**Table S4**), suggesting that the stalk crushing strength should be affected by the physiological morphology of sugarcane stalks. Besides, the frequency distribution statistics showed that stalk crushing strength exhibited a normal distribution in all three planting plots (**Figure 2D**), implying that stalk crushing strength of sugarcane should be a quantitative trait. Notably, upon a correlation analysis of the stalk crushing strengths between the three planting plots, a highly significant (*P* < 0.001) correlation was observed (**Figure 2D**), indicating that stalk crushing strength should be a genetically controlled characteristic that could be stably applied for stalk mechanical strength characterization in sugarcane. Therefore, the observed genetically stable large variation of crushing strengths in the collected sugarcane germplasm population allows for reliable NIR modeling and applications.

### Characterization of sugarcane samples by online near-infrared spectroscopy

**Figure 3:**
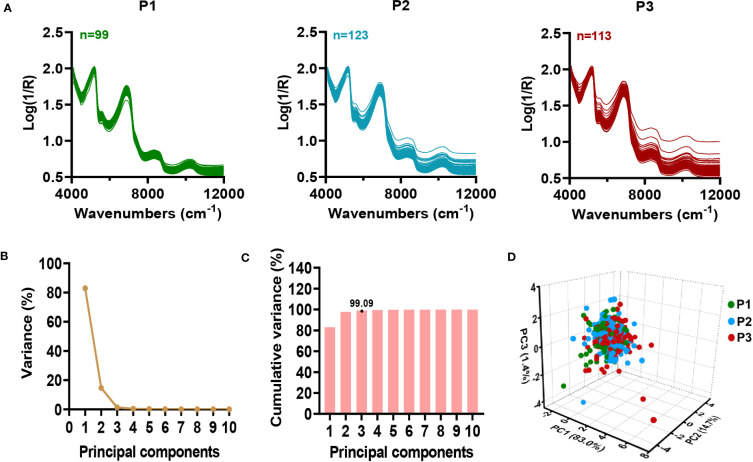
Characterization of near-infrared spectral in 335 sugarcane samples. **(A)** Original spectra of sugarcane samples in three planting plots. **(B-D)** Principal component analysis of NIRS data. **(B)** Contribution of each principal component to variable explanation. **(C)** Cumulative contribution of principal components to variable explanation. **(D)** 3D score view of sugarcane samples by PCA. P1-P3: three planting plots.

A total of 335 sugarcane samples from three planting plots were used for NIRS modeling. The NIR spectral data of collected sugarcane samples showed continuous fluctuations with a normal range (**Figure 3A**), indicating a high level of genetic diversity in the germplasm for biochemical traits (Wang et al., 2021). The principal component analysis (PCA) was employed to identify and classify the samples based on their spectrum (Martin-Tornero et al., 2020). To characterize 335 sugarcane samples, the first ten principal components were extracted from the raw NIRS data. Notably, the first three principal components showed a greater contribution rate to the variable explanation (**Figure 3B**; **Table S5**), which explained 99.09% of the variance (**Figure 3C**; **Table S5**). As a means of better observing the distribution of the samples, the first three principal components were selected in order to generate the 3D scores plot of all samples. Consequently, we observed a relatively symmetrical distribution of sugarcane samples from different planting plots in the 3D plot (**Figure 3D**), with no obvious differences between the planting plots. According to the results, a quantitative analysis model for stalk crushing strength of sugarcane can be developed using online NIRS.

### Online NIRS modeling for stalk crushing strength in sugarcane

**Figure 4:**
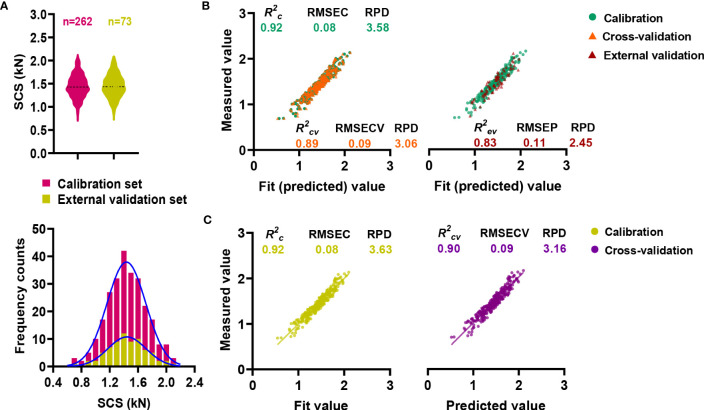
Online NIRS modeling for stalk crushing strength. **(A)** Distribution characteristics of calibration and validation sets. **(B)** Online NIRS calibration and external validation. **(C)** Performance of the integrative final NIRS equation.

In order to ensure accurate and stable NIRS modeling, sugarcane samples were allocated into critical calibration and validation sets (Payne and Wolfrum, 2015). In detail, a total of 262 sugarcane samples were used for calibration, whereas 73 samples were used for external validation. A wide variation range and continuous normal distribution were observed in all samples used for calibration and external validation (**Figure 4A**). Meanwhile, calibration set contained the range of the external validation set (**Table S6**), preventing the predicted value from exceeding the prediction range of model. Since the calibration and external validation sets were comparable, the NIRS model could be calibrated as well as externally validated.

With the assistance of OPUS software, the prediction equation of NIRS model was established through a partial least squares analysis (PLS) (Li et al., 2021). A pretreatment of raw spectral data was carried out before calibration in order to minimize the detrimental effect of the baseline (Devos et al., 2014). A series of complex eliminating modeling processes were applied by the OPUS software to optimize the prediction capability of the obtained equation. Performance of equations were measured by the coefficient of determination (*R^2^*), the root mean square error (RMSE) and the ratio performance deviation (RPD) (Yang et al., 2016). As the calibration result, the coefficient of determination (*R^2^c*) and RPD values were obtained as high as 0.92 and 3.58, respectively (**Figure 4B**; **Table S7**). Besides, cross-validation and external validation of the model were applied for model evaluation, resulting in constant high *R^2^cv*/*R^2^ev* values of over 0.8, RPD values of over 2.4, as well as low root mean square errors of 0.09 and 0.11 kN (**Figure 4B**; **Table S7**).

For the purpose of improving the prediction performance of the equation, external validation and calibration sets were combined to generate the final calibration equation (Windley and Foley, 2015). Although the *R^2^c* value of the equation did not increased significantly during the calibration process (**Figure 4C**; **Table S8**), a higher correlation was observed between the measured value and the predicted value during the internal cross-validation. Accordingly, the *R^2^cv* value increased from 0.89 to 0.90, and the RPD value increased from 3.06 to 3.16 (**Figure 4C**; **Table S8**), indicating that the new equation was capable of making even better predictions.

### Model-based evaluation of stalk crushing strength in sugarcane germplasm

**Figure 5:**
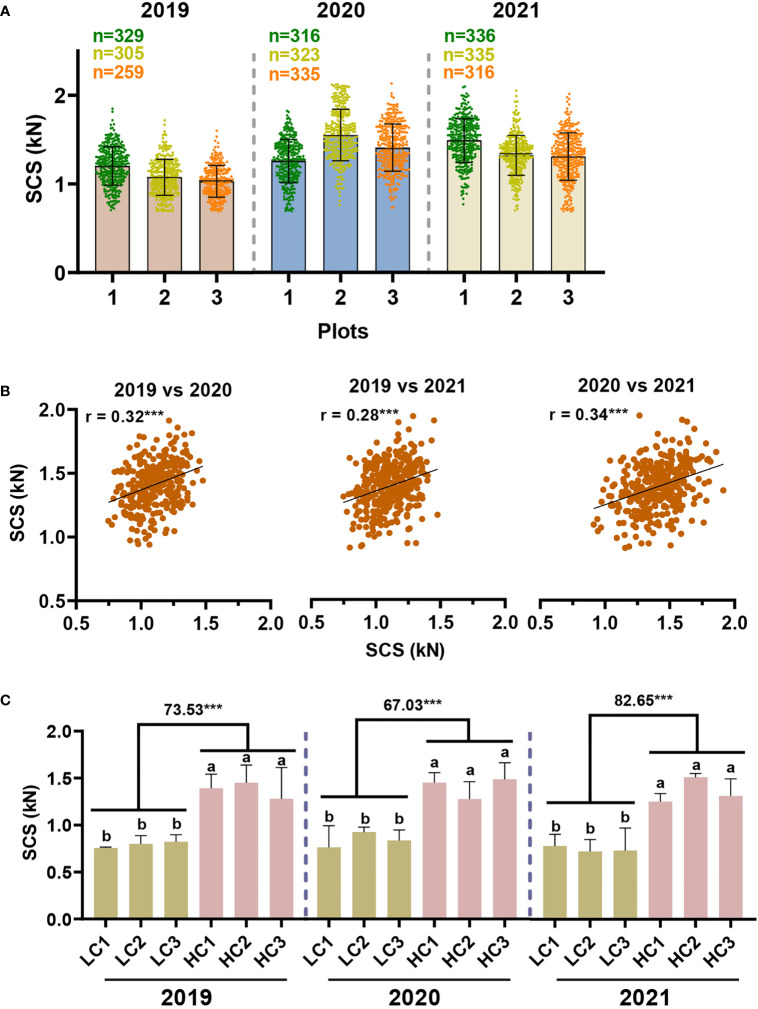
Model-based evaluation of stalk crushing strength in sugarcane germplasm. **(A)** Distribution of stalk crushing strength in sugarcane population. **(B)** Correlation analysis of stalk crushing strength between three years. *** indicated significant correlations at *p* < 0.001 level. **(C)** Comparative analysis of stalk crushing strength in the screened sugarcane germplasm. LC/HC: representing the sugarcane samples with low and high crushing strength, respectively. Different letters indicated statistically significant differences between the groups using one-way ANOVA and LSD test at α ≤ 0.05; *** indicated statistically significant different at *p* < 0.001 levels, respectively.

In an effort to evaluate the performance of our model developed for predicting stalk crushing strength in large-scale sugarcane germplasm, the model was applied to 336 sugarcane genotypes planted in three planting plots over a period of three years (2019, 2020, 2021). As can be seen from the sample data, a limited number of outliers were observed, providing evidence that the model is robust and may be applied widely (**Table S9**). In either of all three planting plots for the same planting year or across different planting years, stalk crushing strength exhibited a similar range of variation (**Figure 5A**). In 2019, the sugarcane population appeared to have a slight lower in mean value and a substantial variation in crushing strength, which may be a result of the rainy climate in that year. Specifically, in 2020 and 2021, stalk crushing strengths were observed ranging from 0.69-2.13 kN and 0.69-2.10 kN, respectively, whereas in 2019 they ranged between 0.69-1.85 kN (**Figure 5A**).

Moreover, we conducted a correlation analysis of the predicted crushing strength of sugarcane samples across different years. It was observed that stalk crushing strength was highly correlated across all three years at a *p* < 0.001 significant level (**Figure 5B**), confirming the findings in NIRS calibration sets (**Figure 2D**), demonstrating that sugarcane stalk crushing strength should have a steady inheritance pattern. It also proved that the model-based characterization of sugarcane crushing strength by means of NIRS is both accurate and stable. Accordingly, sugarcane germplasm with high and low stalk crushing strengths were successfully screened out according to the results predicted by the model. Notably, these screened sugarcane genotypes maintained significant differences in stalk crushing strengths over three years, consistent with the correlation results, further demonstrating that the NIRS model-based method of sugarcane crushing strength analysis is accurate and repeatable (**Figure 5C**). Overall, these findings suggest that the model is practical and can be used to rapidly identify ideal sugarcane germplasm from large-scale populations of sugarcane.

## Discussion

Lodging is one of the major problems that affect the growth and potential yield of agricultural crops (Guo et al., 2021). However, it is challenging to accurately identify the lodging resistance of crops as it is a complex trait affected by a variety of factors (Khobra et al., 2019; Shah et al., 2019). Stem mechanical properties indicate the load-bearing capacity of plants, and therefore can be used as an indirect criterion for selecting lodging-resistant varieties (Yang et al., 2020). Studies have evaluated lodging resistance of stems by measuring their mechanical strength, and it has been found that improving the mechanical strength of stems can reduce lodging risks (Xue et al., 2016; Zhan et al., 2022). In spite of this, it remains a serious problem that there is currently no method of measuring mechanical strength in crops that is both accurate and high-throughput. In our latest study, a precise and high-throughput mechanical strength characterization assay was developed in terms of measuring rind penetrometer resistance (RPR) and breaking force by NIRS modeling in sugarcane (Shen et al., 2022), providing a framework for high-throughput phenotyping of crop stalk mechanical properties. Through a combination of complementary explorations, this study aimed to establish high-throughput phenotyping methods for sugarcane crushing strength.

As a first step toward an effective NIRS calibration, a laboratory analytical method was performed in an effort to ensure accuracy. Owing to an electronic universal testing machine packed with TestExpert software, we were able to obtain the curves of the changes in mechanical properties of sugarcane during crushing (**Figure S1**; **Figure 1D**). A comparative analysis revealed that the first peak of the sugarcane crushing mechanics curve provides a stable assessment of the sugarcane crushing strength (**Figures 1E, F**; **Figure S2**), which was consistent with the findings in maize (Xu et al., 2017). According to our established laboratory analysis method for sugarcane stalk crushing strength determination, a collection of 306 sugarcane germplasms revealed considerable genetic variability (**Figure 2**), which provides a significant basis for NIRS modeling. It should be noted that a total of 416 sugarcane genotypes planted in three test plots (with some samples missing in each plot) were tested for crushing strength characterization (**Figure 2**). However, only 335 of those that showed the best biological replicates across three test plots were selected for NIRS modelling to ensure an accurate calibration (**Figure 3**). As we expected, a high-performance NIRS model for sugarcane crushing strength characterization was obtained based on our established NIRS modeling method. In particular, the model exhibits stable and reliable prediction parameters in both internal cross-validation and external tests (**Figure 4**), indicating excellent performance in practice. In spite of this, it may be possible to improve the model further by adding more reliable data.

Besides, the model was applied to a large-scale phenotypic analysis of sugarcane crushing strength over a period of three consecutive years. Accordingly, the model demonstrated good robustness in its application, with only a few samples being detected as out of range (**Table S9**). In particular, we found that model-based predictions of sugarcane crushing strength showed significant correlations between years (**Figure 5B**). Material with high and low crushing strengths were consistently screened out from sugarcane population for three consecutive years (**Figure 5C**). It was further confirmed that the developed model has excellent predictive performance and can be applied to high-throughput phenotyping of sugarcane crushing strength in sugarcane population.

Notably, in our study, significant correlations were found between sugarcane crushing strength and internode length, stem diameter, and internode number (**Table S4**). This suggests that sugarcane crushing strength is a complex trait that is closely related to the biological properties of sugarcane stalks. This is different from the pattern of rind penetrometer resistance (RPR) and breaking force that characterized in our previous study (Shen et al., 2022). It implies that the application of a single indicator in assessing the mechanical strength of sugarcane stalks to determine the lodging resistant is not desirable. Therefore, the high-throughput phenotypic analysis assay for sugarcane crushing strength determination established in this study, combined with our previously established rapid assays for sugarcane rind penetrometer resistance (RPR) and breaking force characterization (Shen et al., 2021; Shen et al., 2022), can provide a more comprehensive and systematic technical support for lodging resistance targeted sugarcane breeding and beyond.

## Conclusions

Using the established accurate laboratory method for crushing strength characterization as well as effective NIR modeling, this study developed a precise and high-throughput phenotyping assay for the determination of mechanical strength in sugarcane. The obtained final equation *via* integrative modeling exhibited a coefficient of determination (*R^2^*) and ratio performance deviation (RPD) as high as over 0.9 and 3.0, respectively, reflecting excellent prediction capacity. Model-based application provided a stable and effective approach for crushing strength trait evaluation in large-scale sugarcane germplasm screening tasks. This study suggests that the NIRS assay could be applied as a highly reliable tool for lodging-resistant targeted phenotyping jobs.

## Data availability statement

The raw data supporting the conclusions of this article will be made available by the authors, without undue reservation.

## Author contributions

FM completed the major experiment, analyzed the data, and completed the first draft of manuscript. YS and DS participated in crushing strength determination. MW, FJ, MA, QH, XC, GH participated in sugarcane samples preparation and NIRS data collection. WY revised the manuscript. JH and MZ designed the project, supervised the experiments, interpreted the data, and finalized the manuscript. All authors contributed to the article and approved the submitted version.
