# Supplementary material for: A high-throughput phenotyping assay for precisely determining stalk crushing strength in large-scale sugarcane germplasm
Source: Front Plant Sci. 2023 Jul 20;14:1224268. doi: 10.3389/fpls.2023.1224268 (PMC10399216; doi:10.3389/fpls.2023.1224268)
Supplement: Supplementary file 1 [file Presentation_1.pptx]

## Slide 1
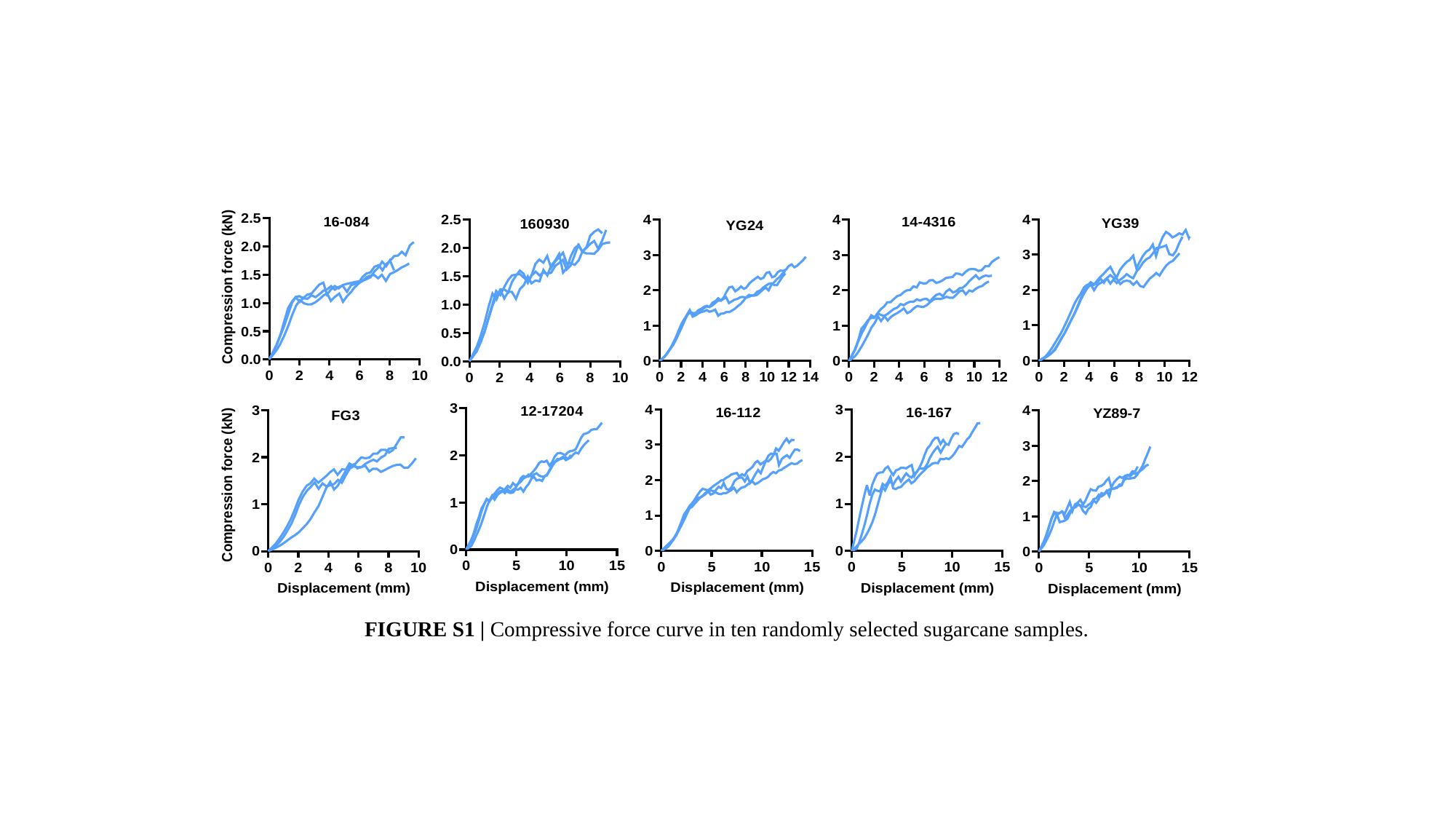

Figure S1 | Compressive force curve in ten randomly selected sugarcane samples.

## Slide 2
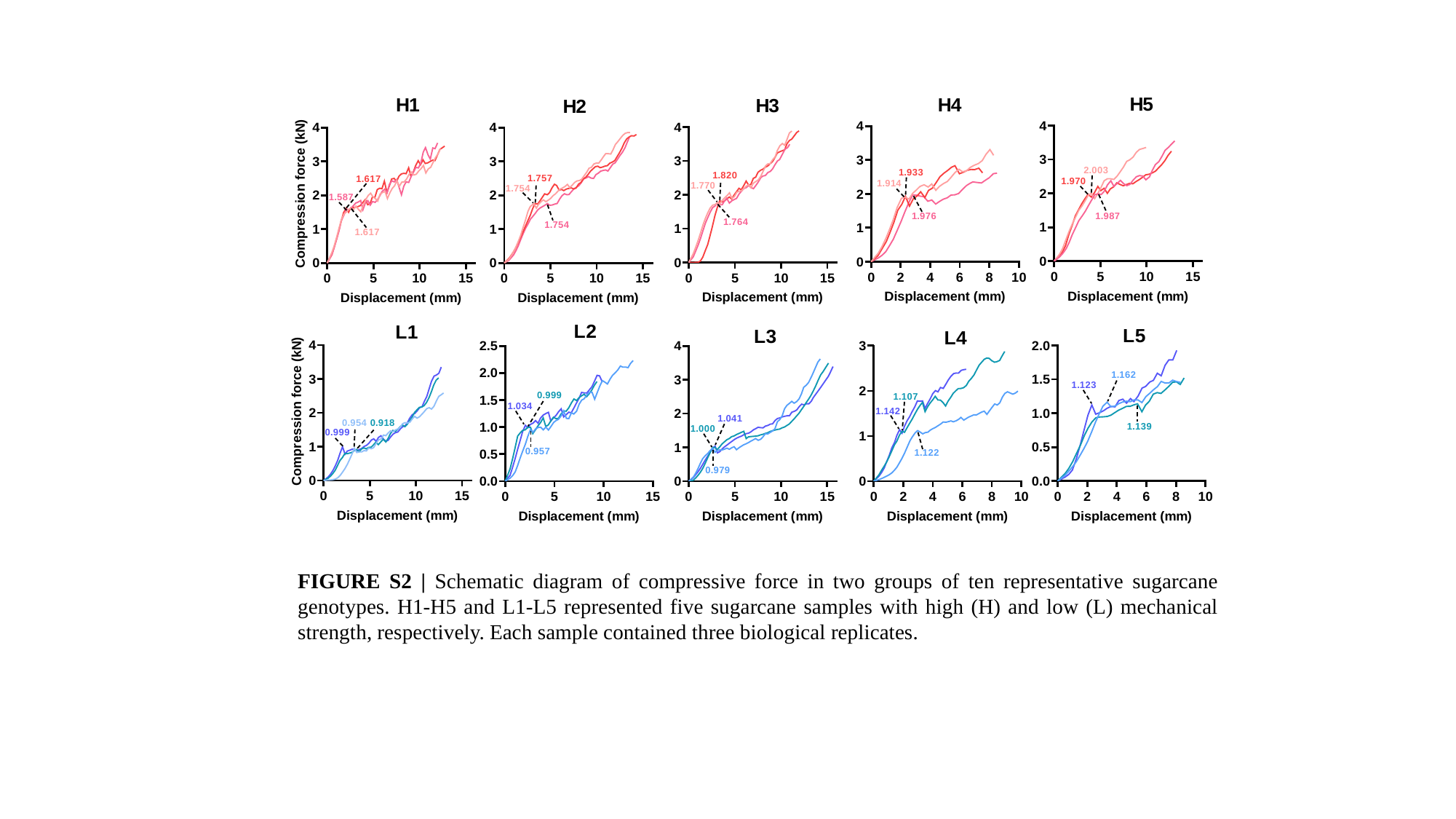

Figure S2 | Schematic diagram of compressive force in two groups of ten representative sugarcane genotypes. H1-H5 and L1-L5 represented five sugarcane samples with high (H) and low (L) mechanical strength, respectively. Each sample contained three biological replicates.

## Slide 3
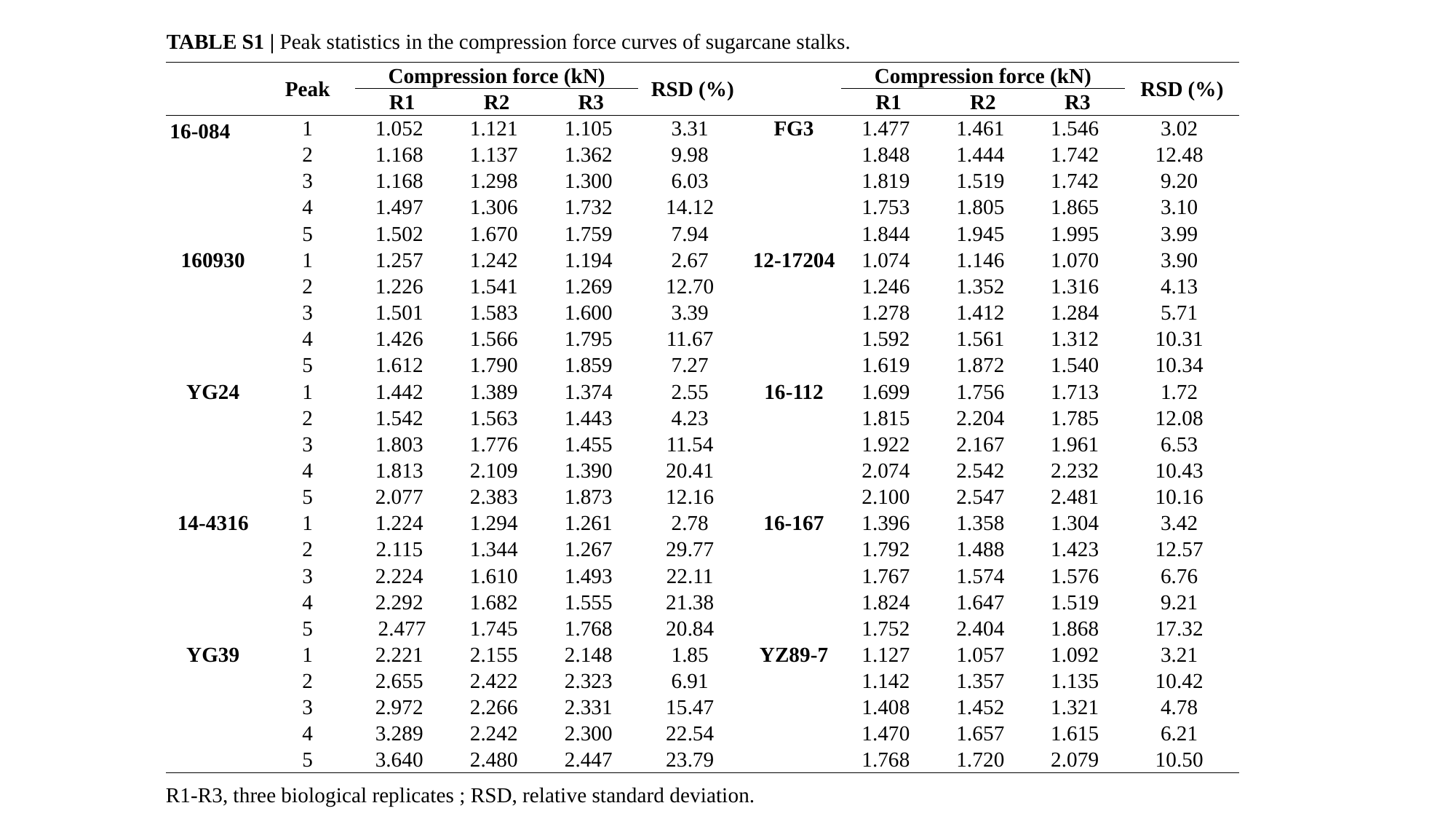

Table S1 | Peak statistics in the compression force curves of sugarcane stalks.
| | Peak | Compression force (kN) | | | RSD (%) | | Compression force (kN) | | | RSD (%) |
| --- | --- | --- | --- | --- | --- | --- | --- | --- | --- | --- |
| | | R1 | R2 | R3 | | | R1 | R2 | R3 | |
| 16-084 | 1 | 1.052 | 1.121 | 1.105 | 3.31 | FG3 | 1.477 | 1.461 | 1.546 | 3.02 |
| | 2 | 1.168 | 1.137 | 1.362 | 9.98 | | 1.848 | 1.444 | 1.742 | 12.48 |
| | 3 | 1.168 | 1.298 | 1.300 | 6.03 | | 1.819 | 1.519 | 1.742 | 9.20 |
| | 4 | 1.497 | 1.306 | 1.732 | 14.12 | | 1.753 | 1.805 | 1.865 | 3.10 |
| | 5 | 1.502 | 1.670 | 1.759 | 7.94 | | 1.844 | 1.945 | 1.995 | 3.99 |
| 160930 | 1 | 1.257 | 1.242 | 1.194 | 2.67 | 12-17204 | 1.074 | 1.146 | 1.070 | 3.90 |
| | 2 | 1.226 | 1.541 | 1.269 | 12.70 | | 1.246 | 1.352 | 1.316 | 4.13 |
| | 3 | 1.501 | 1.583 | 1.600 | 3.39 | | 1.278 | 1.412 | 1.284 | 5.71 |
| | 4 | 1.426 | 1.566 | 1.795 | 11.67 | | 1.592 | 1.561 | 1.312 | 10.31 |
| | 5 | 1.612 | 1.790 | 1.859 | 7.27 | | 1.619 | 1.872 | 1.540 | 10.34 |
| YG24 | 1 | 1.442 | 1.389 | 1.374 | 2.55 | 16-112 | 1.699 | 1.756 | 1.713 | 1.72 |
| | 2 | 1.542 | 1.563 | 1.443 | 4.23 | | 1.815 | 2.204 | 1.785 | 12.08 |
| | 3 | 1.803 | 1.776 | 1.455 | 11.54 | | 1.922 | 2.167 | 1.961 | 6.53 |
| | 4 | 1.813 | 2.109 | 1.390 | 20.41 | | 2.074 | 2.542 | 2.232 | 10.43 |
| | 5 | 2.077 | 2.383 | 1.873 | 12.16 | | 2.100 | 2.547 | 2.481 | 10.16 |
| 14-4316 | 1 | 1.224 | 1.294 | 1.261 | 2.78 | 16-167 | 1.396 | 1.358 | 1.304 | 3.42 |
| | 2 | 2.115 | 1.344 | 1.267 | 29.77 | | 1.792 | 1.488 | 1.423 | 12.57 |
| | 3 | 2.224 | 1.610 | 1.493 | 22.11 | | 1.767 | 1.574 | 1.576 | 6.76 |
| | 4 | 2.292 | 1.682 | 1.555 | 21.38 | | 1.824 | 1.647 | 1.519 | 9.21 |
| | 5 | 2.477 | 1.745 | 1.768 | 20.84 | | 1.752 | 2.404 | 1.868 | 17.32 |
| YG39 | 1 | 2.221 | 2.155 | 2.148 | 1.85 | YZ89-7 | 1.127 | 1.057 | 1.092 | 3.21 |
| | 2 | 2.655 | 2.422 | 2.323 | 6.91 | | 1.142 | 1.357 | 1.135 | 10.42 |
| | 3 | 2.972 | 2.266 | 2.331 | 15.47 | | 1.408 | 1.452 | 1.321 | 4.78 |
| | 4 | 3.289 | 2.242 | 2.300 | 22.54 | | 1.470 | 1.657 | 1.615 | 6.21 |
| | 5 | 3.640 | 2.480 | 2.447 | 23.79 | | 1.768 | 1.720 | 2.079 | 10.50 |
R1-R3, three biological replicates ; RSD, relative standard deviation.

## Slide 4
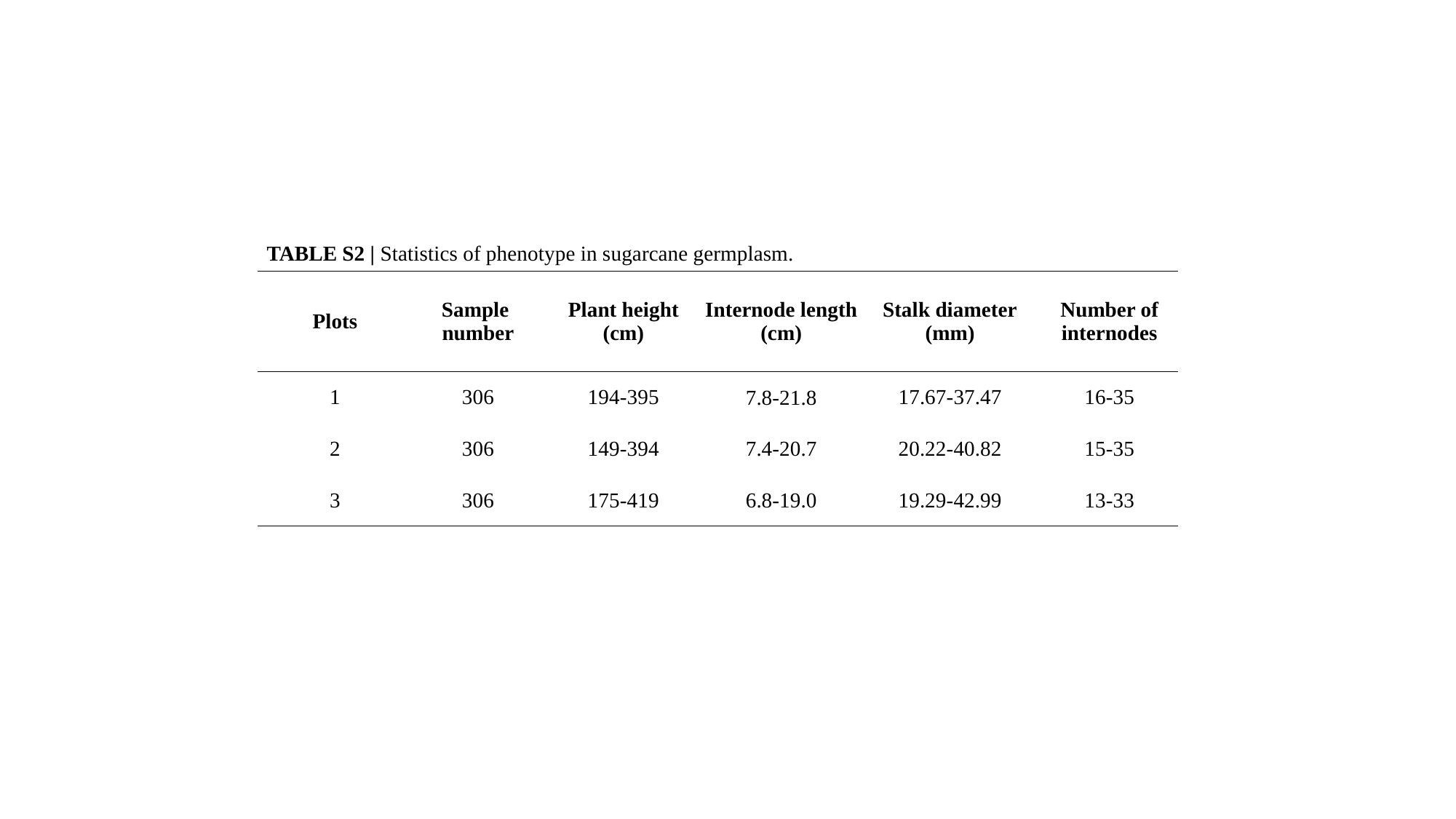

Table S2 | Statistics of phenotype in sugarcane germplasm.
| Plots | Sample number | Plant height (cm) | Internode length (cm) | Stalk diameter (mm) | Number of internodes |
| --- | --- | --- | --- | --- | --- |
| 1 | 306 | 194-395 | 7.8-21.8 | 17.67-37.47 | 16-35 |
| 2 | 306 | 149-394 | 7.4-20.7 | 20.22-40.82 | 15-35 |
| 3 | 306 | 175-419 | 6.8-19.0 | 19.29-42.99 | 13-33 |

## Slide 5
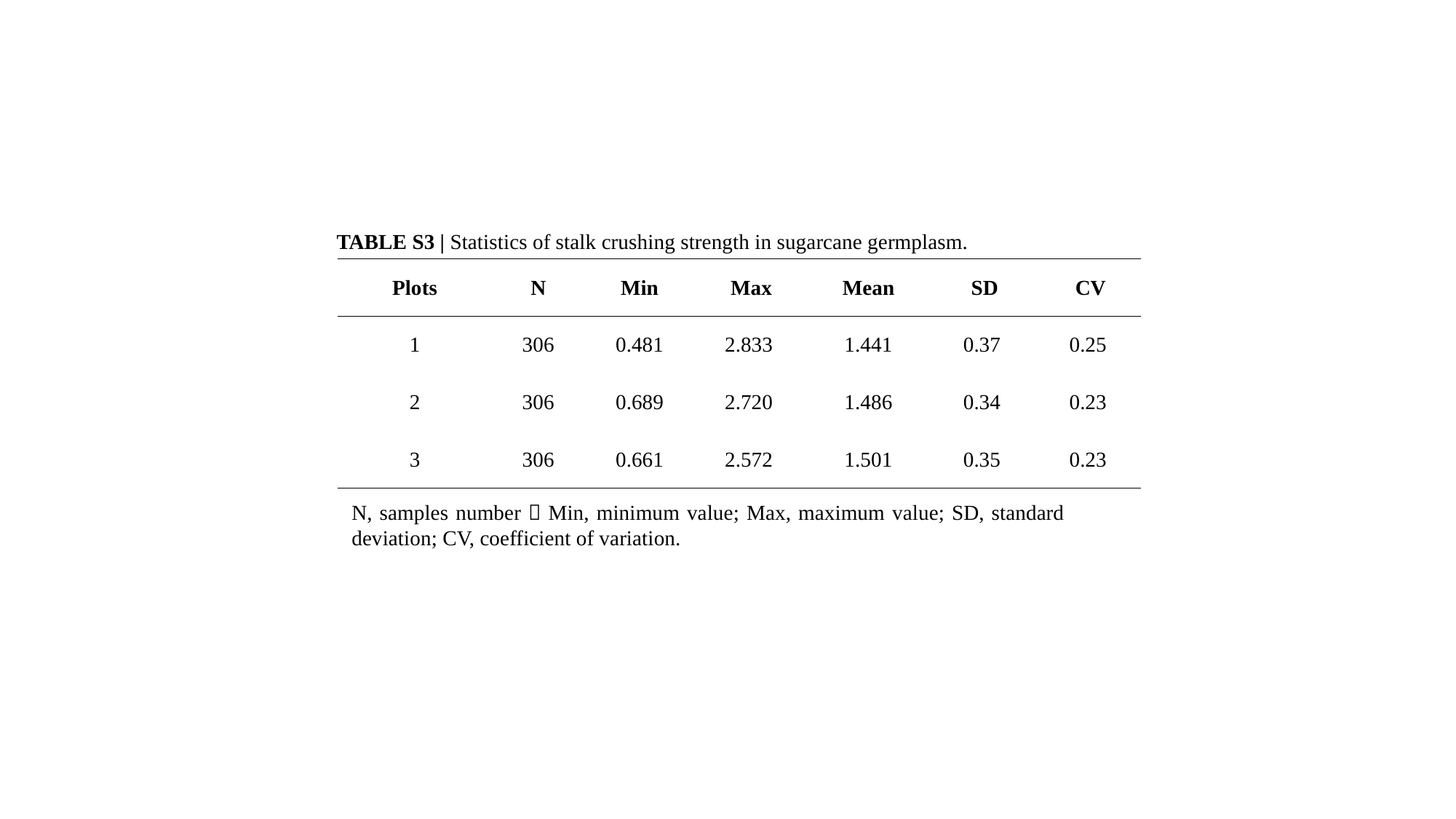

Table S3 | Statistics of stalk crushing strength in sugarcane germplasm.
| Plots | N | Min | Max | Mean | SD | CV |
| --- | --- | --- | --- | --- | --- | --- |
| 1 | 306 | 0.481 | 2.833 | 1.441 | 0.37 | 0.25 |
| 2 | 306 | 0.689 | 2.720 | 1.486 | 0.34 | 0.23 |
| 3 | 306 | 0.661 | 2.572 | 1.501 | 0.35 | 0.23 |
N, samples number；Min, minimum value; Max, maximum value; SD, standard deviation; CV, coefficient of variation.

## Slide 6
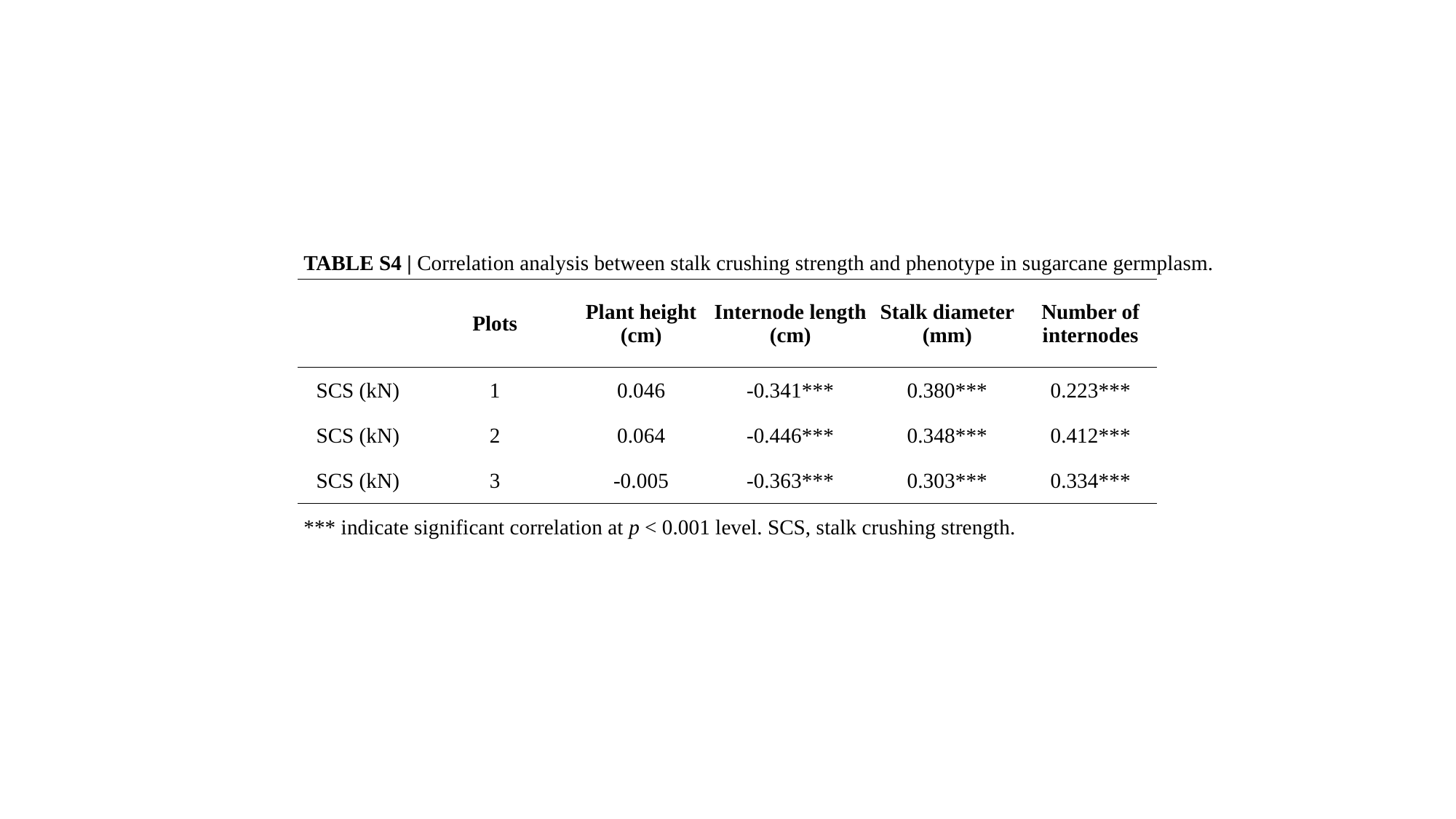

Table S4 | Correlation analysis between stalk crushing strength and phenotype in sugarcane germplasm.
| | Plots | Plant height (cm) | Internode length (cm) | Stalk diameter (mm) | Number of internodes |
| --- | --- | --- | --- | --- | --- |
| SCS (kN) | 1 | 0.046 | -0.341\*\*\* | 0.380\*\*\* | 0.223\*\*\* |
| SCS (kN) | 2 | 0.064 | -0.446\*\*\* | 0.348\*\*\* | 0.412\*\*\* |
| SCS (kN) | 3 | -0.005 | -0.363\*\*\* | 0.303\*\*\* | 0.334\*\*\* |
*** indicate significant correlation at p < 0.001 level. SCS, stalk crushing strength.

## Slide 7
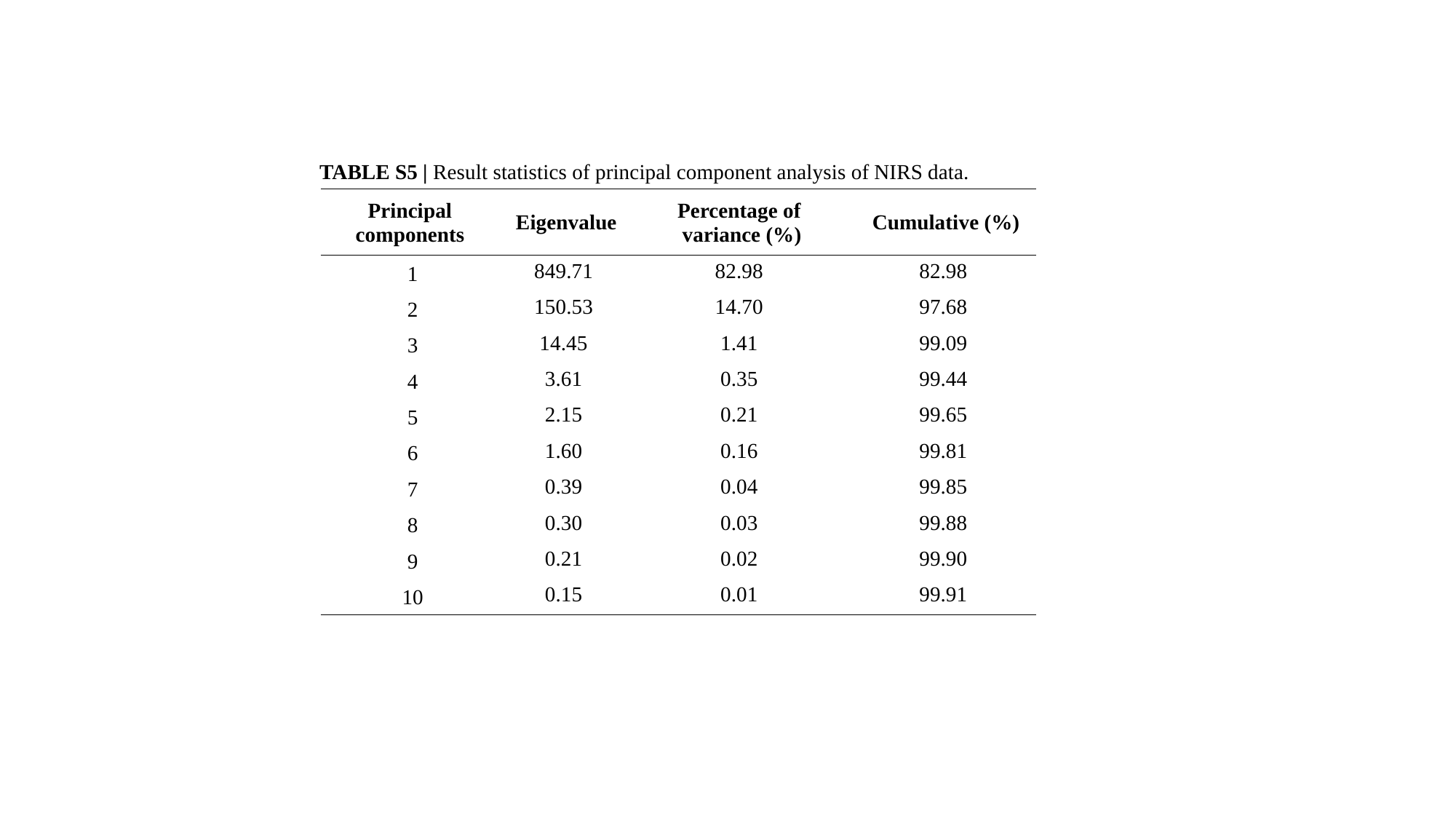

Table S5 | Result statistics of principal component analysis of NIRS data.
| Principal components | Eigenvalue | Percentage of variance (%) | Cumulative (%) |
| --- | --- | --- | --- |
| 1 | 849.71 | 82.98 | 82.98 |
| 2 | 150.53 | 14.70 | 97.68 |
| 3 | 14.45 | 1.41 | 99.09 |
| 4 | 3.61 | 0.35 | 99.44 |
| 5 | 2.15 | 0.21 | 99.65 |
| 6 | 1.60 | 0.16 | 99.81 |
| 7 | 0.39 | 0.04 | 99.85 |
| 8 | 0.30 | 0.03 | 99.88 |
| 9 | 0.21 | 0.02 | 99.90 |
| 10 | 0.15 | 0.01 | 99.91 |

## Slide 8
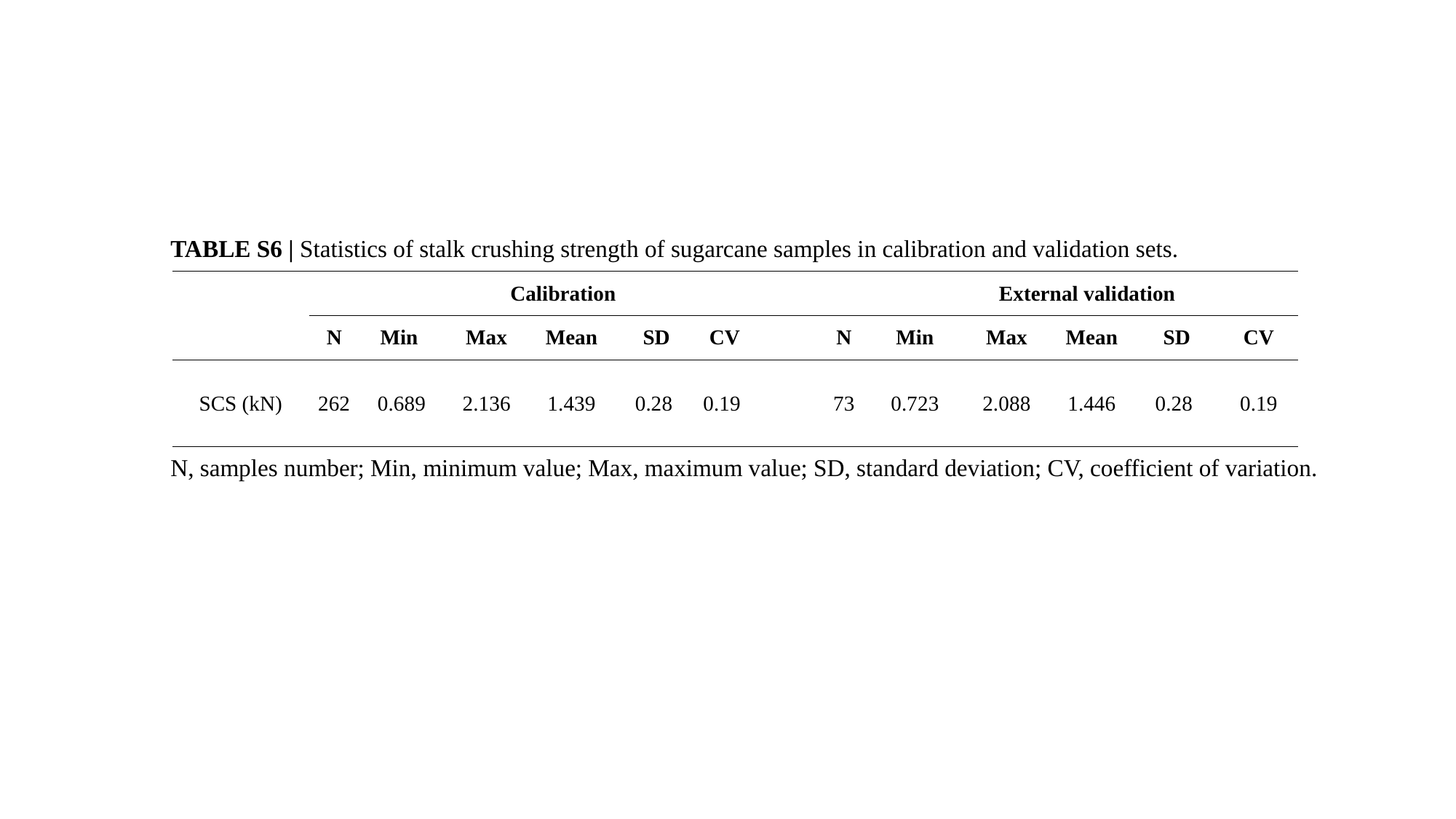

Table S6 | Statistics of stalk crushing strength of sugarcane samples in calibration and validation sets.
| | Calibration | | | | | | | | External validation | | | | |
| --- | --- | --- | --- | --- | --- | --- | --- | --- | --- | --- | --- | --- | --- |
| | N | Min | Max | Mean | SD | CV | | N | Min | Max | Mean | SD | CV |
| SCS (kN) | 262 | 0.689 | 2.136 | 1.439 | 0.28 | 0.19 | | 73 | 0.723 | 2.088 | 1.446 | 0.28 | 0.19 |
N, samples number; Min, minimum value; Max, maximum value; SD, standard deviation; CV, coefficient of variation.

## Slide 9
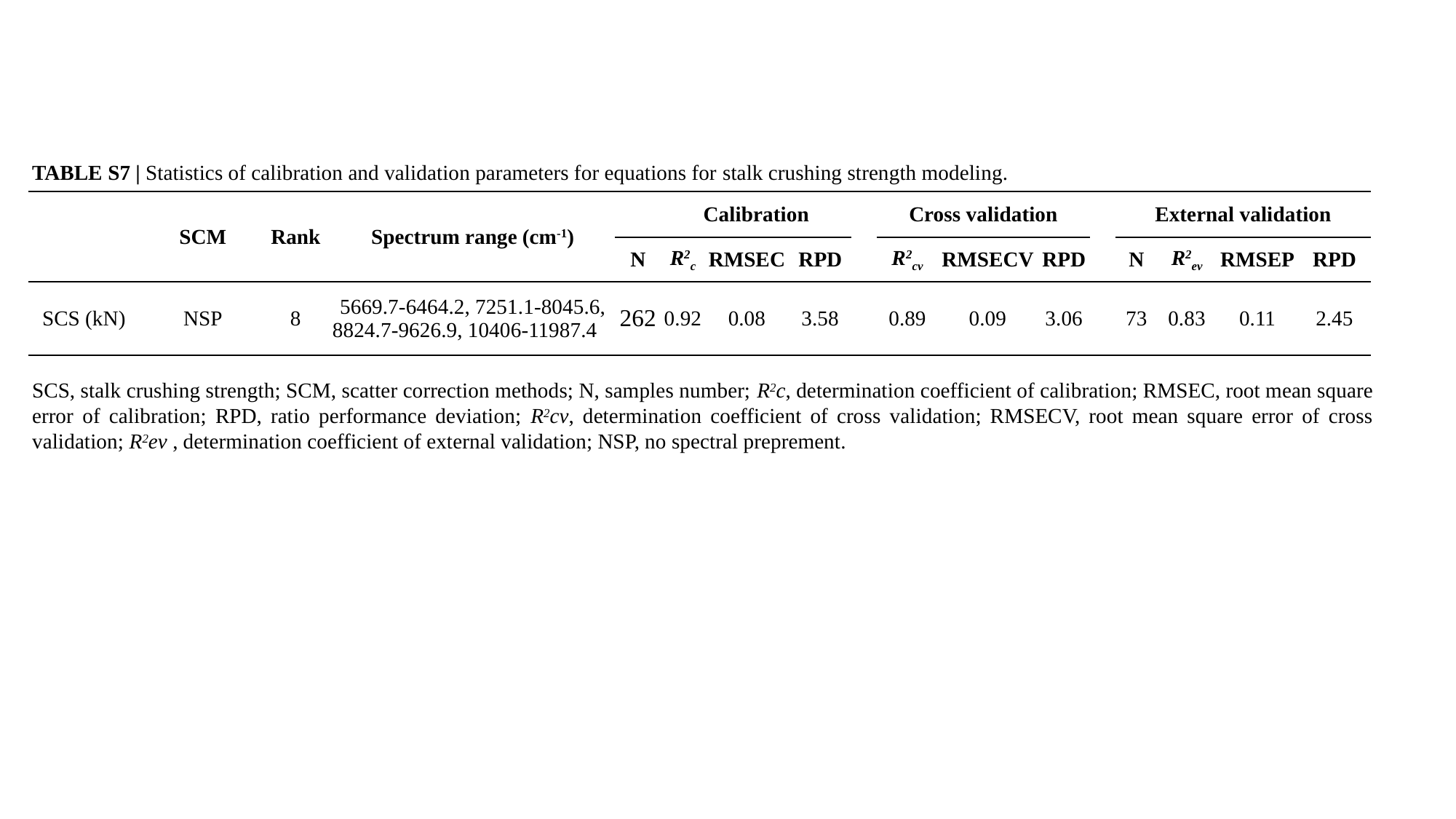

Table S7 | Statistics of calibration and validation parameters for equations for stalk crushing strength modeling.
| | SCM | Rank | Spectrum range (cm-1) | | Calibration | | | | Cross validation | | | | External validation | | | |
| --- | --- | --- | --- | --- | --- | --- | --- | --- | --- | --- | --- | --- | --- | --- | --- | --- |
| | | | | N | R2c | RMSEC | RPD | | R2cv | RMSECV | RPD | | N | R2ev | RMSEP | RPD |
| SCS (kN) | NSP | 8 | 5669.7-6464.2, 7251.1-8045.6, 8824.7-9626.9, 10406-11987.4 | 262 | 0.92 | 0.08 | 3.58 | | 0.89 | 0.09 | 3.06 | | 73 | 0.83 | 0.11 | 2.45 |
SCS, stalk crushing strength; SCM, scatter correction methods; N, samples number; R2c, determination coefficient of calibration; RMSEC, root mean square error of calibration; RPD, ratio performance deviation; R2cv, determination coefficient of cross validation; RMSECV, root mean square error of cross validation; R2ev , determination coefficient of external validation; NSP, no spectral preprement.

## Slide 10
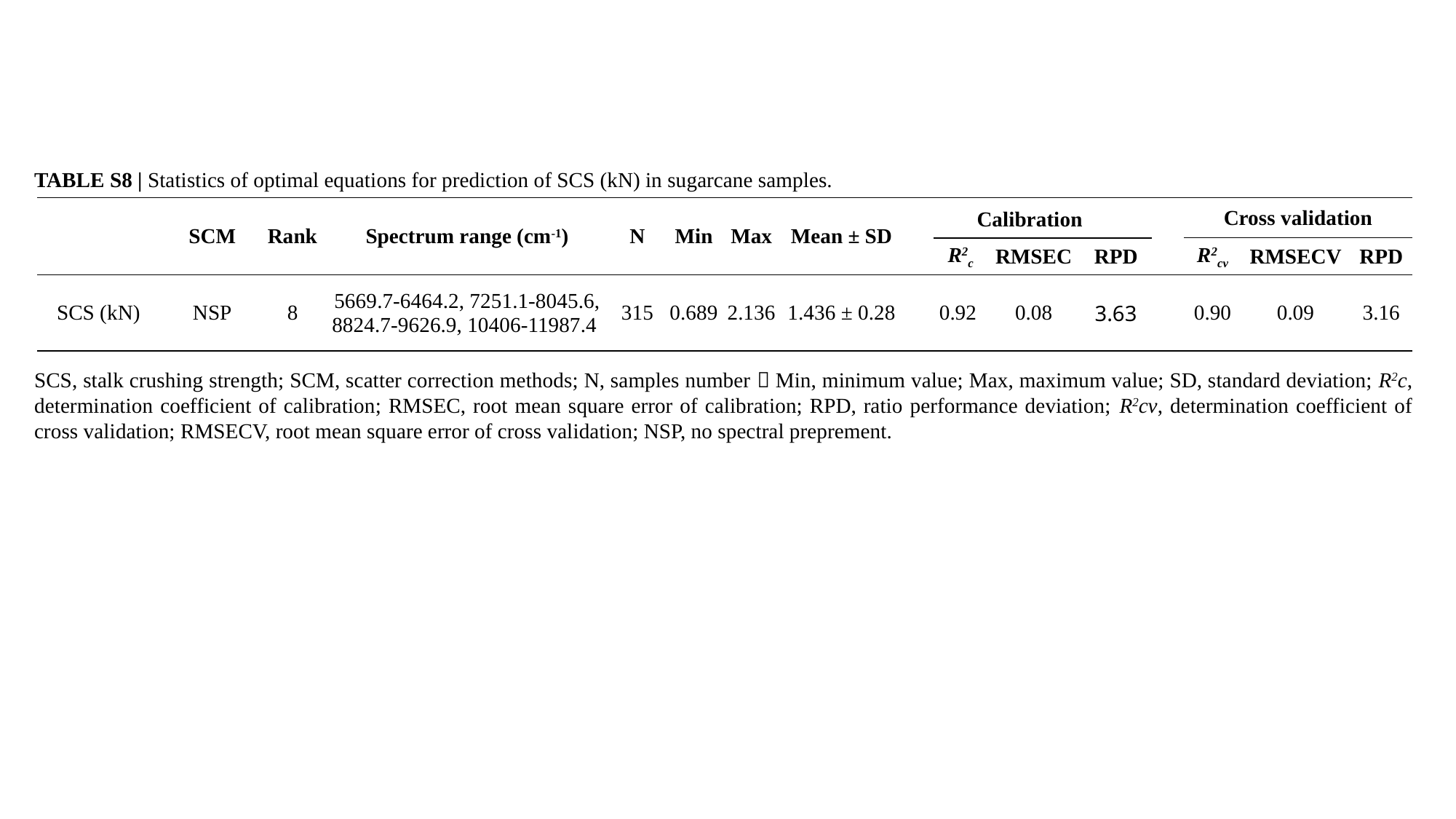

Table S8 | Statistics of optimal equations for prediction of SCS (kN) in sugarcane samples.
| | SCM | Rank | Spectrum range (cm-1) | N | Min | Max | Mean ± SD | | Calibration | | | | Cross validation | | |
| --- | --- | --- | --- | --- | --- | --- | --- | --- | --- | --- | --- | --- | --- | --- | --- |
| | | | | | | | | | R2c | RMSEC | RPD | | R2cv | RMSECV | RPD |
| SCS (kN) | NSP | 8 | 5669.7-6464.2, 7251.1-8045.6, 8824.7-9626.9, 10406-11987.4 | 315 | 0.689 | 2.136 | 1.436 ± 0.28 | | 0.92 | 0.08 | 3.63 | | 0.90 | 0.09 | 3.16 |
SCS, stalk crushing strength; SCM, scatter correction methods; N, samples number；Min, minimum value; Max, maximum value; SD, standard deviation; R2c, determination coefficient of calibration; RMSEC, root mean square error of calibration; RPD, ratio performance deviation; R2cv, determination coefficient of cross validation; RMSECV, root mean square error of cross validation; NSP, no spectral preprement.

## Slide 11
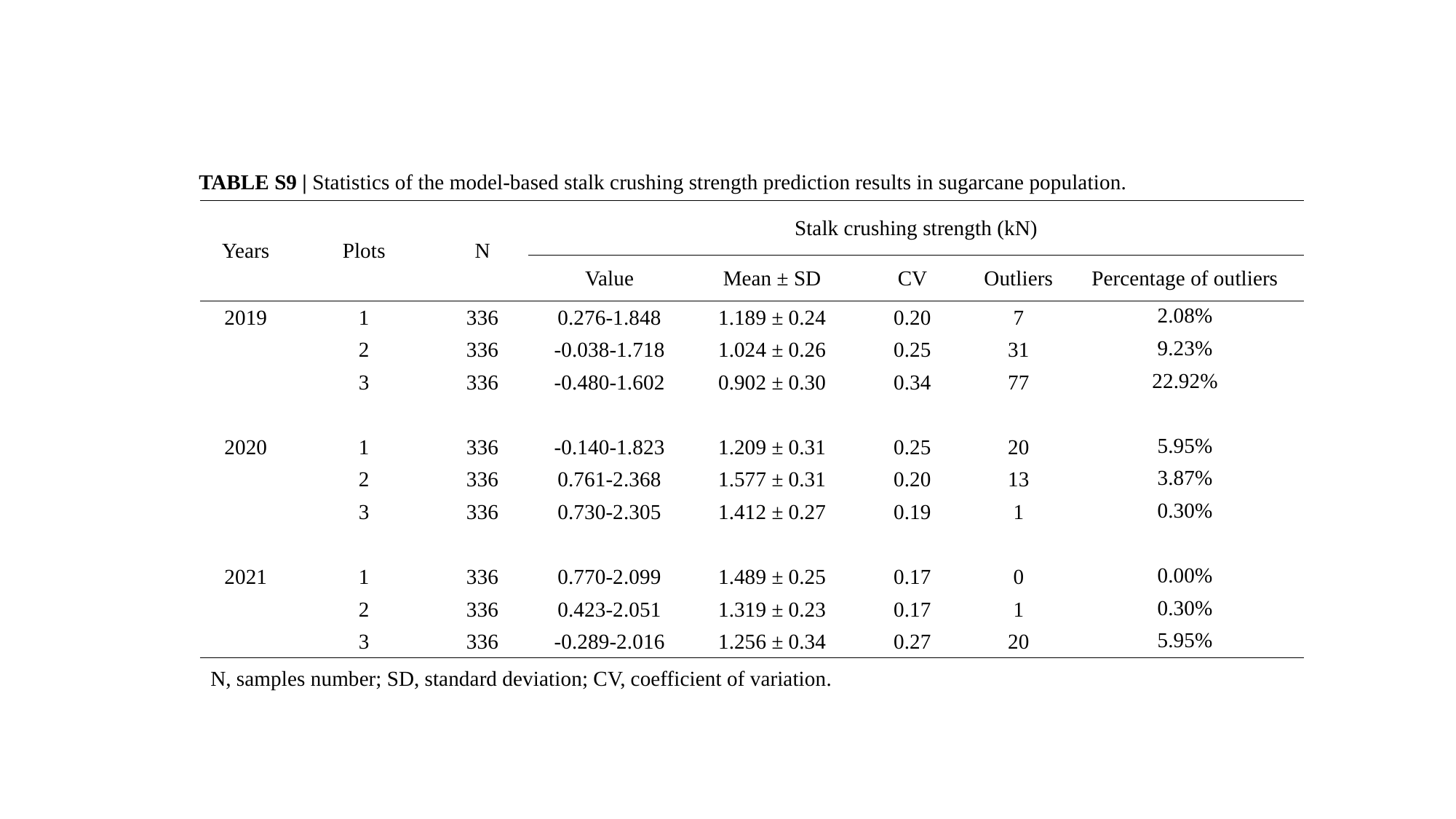

Table S9 | Statistics of the model-based stalk crushing strength prediction results in sugarcane population.
| Years | Plots | N | Stalk crushing strength (kN) | | | | |
| --- | --- | --- | --- | --- | --- | --- | --- |
| | | | Value | Mean ± SD | CV | Outliers | Percentage of outliers |
| 2019 | 1 | 336 | 0.276-1.848 | 1.189 ± 0.24 | 0.20 | 7 | 2.08% |
| | 2 | 336 | -0.038-1.718 | 1.024 ± 0.26 | 0.25 | 31 | 9.23% |
| | 3 | 336 | -0.480-1.602 | 0.902 ± 0.30 | 0.34 | 77 | 22.92% |
| | | | | | | | |
| 2020 | 1 | 336 | -0.140-1.823 | 1.209 ± 0.31 | 0.25 | 20 | 5.95% |
| | 2 | 336 | 0.761-2.368 | 1.577 ± 0.31 | 0.20 | 13 | 3.87% |
| | 3 | 336 | 0.730-2.305 | 1.412 ± 0.27 | 0.19 | 1 | 0.30% |
| | | | | | | | |
| 2021 | 1 | 336 | 0.770-2.099 | 1.489 ± 0.25 | 0.17 | 0 | 0.00% |
| | 2 | 336 | 0.423-2.051 | 1.319 ± 0.23 | 0.17 | 1 | 0.30% |
| | 3 | 336 | -0.289-2.016 | 1.256 ± 0.34 | 0.27 | 20 | 5.95% |
N, samples number; SD, standard deviation; CV, coefficient of variation.
